# Bloch surface waves confined in one dimension with a single polymeric nanofibre

**DOI:** 10.1038/ncomms14330

**Published:** 2017-02-03

**Authors:** Ruxue Wang, Hongyan Xia, Douguo Zhang, Junxue Chen, Liangfu Zhu, Yong Wang, Erchan Yang, Tianyang Zang, Xiaolei Wen, Gang Zou, Pei Wang, Hai Ming, Ramachandram Badugu, Joseph R. Lakowicz

**Affiliations:** 1 Department of Optics and Optical Engineering, Institute of Photonics, University of Science and Technology of China, Number 96 Jinzhai Road, Hefei, Anhui 230026, China; 2 CAS Key Laboratory of Soft Matter Chemistry, Department of Polymer Science and Engineering, iChEM, University of Science and Technology of China, Hefei, Anhui 230026, China; 3 School of Science, Southwest University of Science and Technology, Mianyang, Sichuan 621010, China; 4 Center for Micro- and Nanoscale Research and Fabrication, Hefei National Laboratory for Physical Sciences at the Microscale, University of Science and Technology of China, Hefei, Anhui 230026, China; 5 Center for Fluorescence Spectroscopy, Department of Biochemistry and Molecular Biology, University of Maryland School of Medicine, Baltimore, Maryland 21201, USA

## Abstract

Polymeric fibres with small radii (such as ≤125 nm) are delicate to handle and should be laid down on a solid substrate to obtain practical devices. However, placing these nanofibres on commonly used glass substrates prevents them from guiding light. In this study, we numerically and experimentally demonstrate that when the nanofibre is placed on a suitable dielectric multilayer, it supports a guided mode, a Bloch surface wave (BSW) confined in one dimension. The physical origin of this new mode is discussed in comparison with the typical two-dimensional BSW mode. Polymeric nanofibres are easily fabricated to contain fluorophores, which make the dielectric nanofibre and multilayer configuration suitable for developing a large range of new nanometric scale devices, such as processor–memory interconnections, devices with sensitivity to target analytes, incident polarization and multi-colour BSW modes.

## Abstract (web summary)

Typically nanofibres need to be placed on solid substrates for the next generation of devices, but this prevents light guiding. Here Wang *et al*. numerically and experimentally demonstrate that when a nanofibre is placed on a dielectric multilayer, it supports a Bloch surface wave confined in one dimension.

In recent years, nanowires or nanofibres made of polymeric, semiconducting or their hybrid materials have attracted a great deal of attention. For example, semiconductor alloy nanowires with spatially graded compositions (and bandgaps) provide a new material platform for many new multifunctional optoelectronic devices, such as broadly tunable lasers, multispectral photo-detectors, broad-band light-emitting diodes and high-efficiency solar cells^1^. Functionalized polymer nanofibres provide a versatile platform for manipulating light on the nanoscale^2^. These applications demonstrate the advantages of dielectric nanofibres (or nanowires) for a wide range of technologies. In the past years, electrospinning has been developed to the point where it is now a rapid and efficient process to fabricate continuous ultra-long one-dimensional (1D) nanomaterials composed of polymers, oxides, carbon and, more recently, metals^3^. Electrospun fibres also display excellent biocompatibility, can be doped, are low cost and are relatively simple to align, assemble and process for a variety of applications^4^. Polymeric nanofibres are fragile, delicate to handle and must be laid down on a solid substrate (such as the commonly used glass substrate), to lead to practical devices. However, nanofibres on glass substrates cannot transport optical signals if the fibre radius is too small. For example, a nanofibre with a radius of 125 nm will not support signals at 632.8 nm wavelength.

Different from the bare glass substrates, periodic dielectric-multilayer substrates contain the well-known photonic band gap (PBG) and, in some cases, support the Bloch surface waves (BSWs). The multilayer substrates can be adopted to support the fragile nanofibres^5^,^6^. Similar to surface plasmon polaritons (SPPs), BSWs are also confined close to the interface between a dielectric multilayer and the surrounding medium, but with somewhat weaker localization; the resonant angle or wavelength (referred to the position of the dip in the reflection spectrum) is very sensitive to the environment^7^,^8^. BSWs also possess specific properties that differentiate them from SPPs. They do not suffer from losses caused by metal absorption that allow for BSW resonances with high-quality factors and enhanced field intensities (*E*^2^)^9^,^10^,^11^. BSWs can be either transverse electric (TE) or transverse magnetic polarized electromagnetic waves. Because of these similarities and differences with SPPs, and the extensive use of surface-bound biomolecules in research and medical diagnostics, in recent years BSWs have received much attention from the research community in recent years. For example, studies have proposed and demonstrated that BSWs have some specific sensing properties, which include chemical and biosensing, gas sensing, fluorescence emission enhancement and sorting, and surface-enhanced Raman scattering^12^,^13^,^14^,^15^,^16^,^17^. The surface confinement of BSWs allows their propagation to be controlled by planar two-dimensional (2D) optical components, such as the controlled propagation of BSWs with simple 2D lenses^18^. Similar applications have also been demonstrated with SPP-based platform. Recently, many 1D configurations have been proposed to function as SPP waveguides, such as metal nano-stripes, V-shaped grooves milled in a metal film and chemically synthesized crystalline-silver nanowires^19^,^20^,^21^,^22^,^23^. These guided SPPs, which are referred to as SPPs with 1D materials (SPP-1D), are attracting great interest for use in high-density photonic nanocircuits. Similar to guiding SPPs with 1D materials, previous reports show that BSWs also can be guided with polymer or silicon ridges (of rectangular shape) fabricated on the top surfaces of dielectric multilayers^24^,^25^,^26^,^27^. This approach results in the shift of an existing BSW to a different resonant angle, so that different locations on the top surface of the multilayer have different resonant angles. The principle behind this kind of BSW waveguide is similar to the dielectric-loaded SPP waveguides where the structure displays two plasmon resonances, one at the metal–air interface and the other at the metal–dielectric interface^28^. The analogous BSW waveguide would also have two resonances and can similarly be referred to as a dielectric-loaded BSW (DLBSW). To the best of our knowledge, no one has yet reported on how to sustain a BSW purely on a 1D dielectric nanostructure that is a structure where there is a single BSW resonance. We show that this can be accomplished with a polymeric nanofibre (of round shape, made with electrospinning) on a dielectric multilayer. Here, optical signals can be transported owing to the formation of a BSW confined in 1D (BSW-1D) and closely confined within the nanofibre, when the nanofibre is placed on a multi-layer substrate. The weaker localization of the BSWs over that of SPPs provides an alternative to the SPPs-based sensing or microscopy, where highly confined plasmonic field is not suitable for sensing or imaging of deeper samples (such as cells). In addition, the metal nanostructures for SPPs are not as biocompatible as that of dielectric structure for BSWs. The most important result is that this BSW-1D mode made the thin polymeric nanofibre transport optical signals even its radius is as small as 125 nm here. The nanofibres can be made from a wide range of dielectric materials and, because of their chemical composition, can easily be doped with fluorophores and other functional units from the outside or inside during manufacture, making them suitable for developing active devices.

## Results

### Properties of a planar waveguide

**Figure 1:**
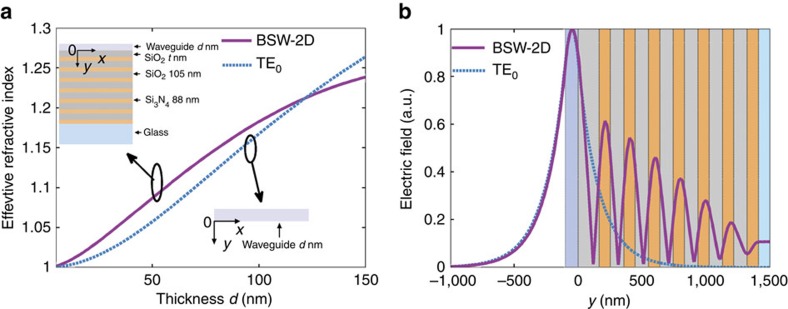
Planar waveguide in air and on a dielectric multilayer. (**a**) The effective refractive indexes versus the thickness of the planar waveguide for the BSW-2D mode sustained by the dielectric multilayer and TE_0_ mode supported by the planar waveguide in air; (**b**) the electric field distributions for the BSW-2D and TE_0_ modes with waveguide thickness *d*=100 nm. The refractive index of waveguide layer is 1.57. The incident wavelength is 632.8 nm. *Y*=0 nm indicates the interface between the multilayer and the planar waveguide.

The properties of a BSW-1D are most easily understood starting from a BSWs' ability to be sustained on a 2D dielectric multilayer. In the inset of Fig. 1a, a planar dielectric waveguide (made of the polymer Nylon-6, which will be used in the following experiments and has a refractive index of 1.57) is shown in air and on a dielectric multilayer for comparison. For this waveguide the wavelength was set to 632.8 nm. This planar waveguide is of infinite width and depth (*X* direction and *Z* direction) and finite thickness (*d* nm, *Y* direction). The refractive index of the glass was 1.515. The dielectric multilayer (14 layers) consisted of alternating layers of SiO_2_ (low refractive index, *n*=1.46+i10^−5^) and Si_3_N_4_ (high refractive index, *n*=2.14+i3 × 10^−4^). The thicknesses of the SiO_2_ and Si_3_N_4_ layers were 105 and 88 nm, respectively. The thickness of the top SiO_2_ layer was chosen to be 165 nm, because BSWs cannot be generated on the dielectric multilayer without a thicker top layer. When this 165 nm planar waveguide is put on the multilayer (Fig. 1a, inset graph, top left corner), optical waves propagating along the *Z*- direction (parallel to the surface and perpendicular to the paper plane) can be generated: they are the well-known BSWs on a 2D plane (BSW-2D). With these structural parameters and for this wavelength of light, only TE-polarized BSWs can be excited. Conversely, when the waveguide is in air (Fig. 1a, inset graph, lower right corner), it can be viewed as a symmetric slab waveguide sustaining both transverse magnetic (TM_0_) and TE_0_ modes without a cutoff thickness (*d*). For comparison with the BSW-2D mode, we only focus on the TE_0_ mode. Effective refractive indexes and electric field distributions for the BSW-2D and TE_0_ mode case are numerically calculated in a rigorous computational analysis based on the finite element method^29^. Figure 1a shows that the effective indexes of both modes increase with thickness *d*. The electric field intensity distributions (along the *Y* axis, where *Y*=0 nm represents the spatial position of the lower surface of the planar waveguide) of the two modes plotted in Fig. 1b. Here, *d* was chosen to be 100 nm and the effective indexes of the BSW-2D and TE_0_ mode were 1.17 and 1.18, respectively. We found that the intensity distributions nearly overlapped inside the waveguide and in air for both modes; in particular, we found that the locations of their peaks were nearly the same (*Y*=−46 nm, inside the planar waveguide). These similarities in the field distributions above the substrate are also preserved for different thicknesses *d* (such as for *d*=50 nm in Supplementary Fig. 1a and *d*=150 nm in Supplementary Fig. 1b).

### Comparison of nanofibre in air and on a dielectric multilayer

**Figure 2:**
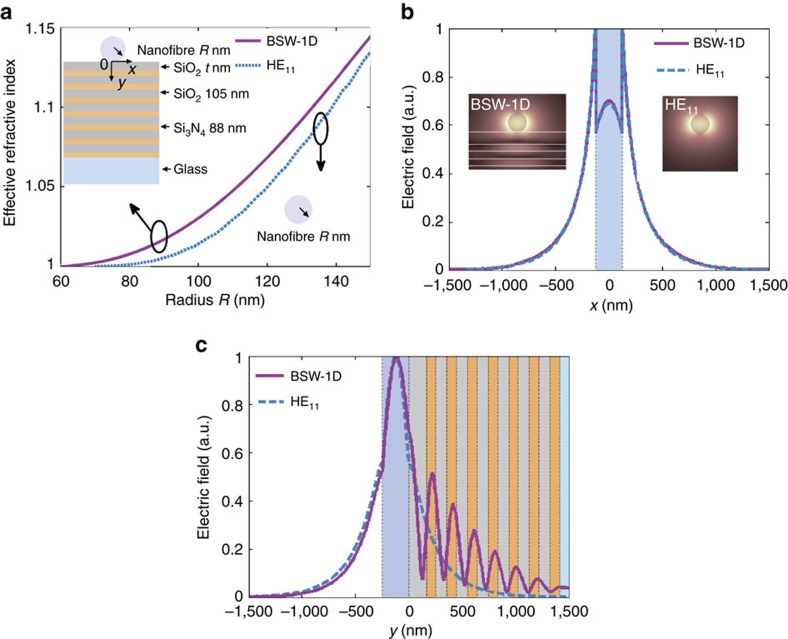
Nanofibre in air and on a multilayer. (**a**) The effective refractive indexes versus the nanofibre radius for the BSW-1D mode guided by the nanofibre on a dielectric multilayer and HE_11_ mode guided by the nanofibre in air. Within the multilayers, the SiO_2_ thickness is 105 nm and the Si_3_N_4_ thickness is 88 nm; (**b**) the electric field distributions for BSW-1D and HE_11_ modes extracted along the horizontal direction of the centre of the nanofibre with radius *R*=125 nm (here, *Y*=−62.5 nm); inset in **b** shows the electric field distribution of the HE_11_ mode and BSW-1D mode with *R*=125 nm. (**c**) The electric field distributions for the BSW-1D and HE_11_ modes extracted along the vertical direction of the centre of the nanofibre with radius *R*=125 nm (here, *X*=0 nm).

When the 2D planar waveguide is exchanged for a 1D nanofibre with a cylindrical shape (inset graphs in Fig. 2a; the long axis of the fibre is along the *z* direction and the selected material is Nylon-6), we see similarities between the modes sustained by the nanofibres in air (Fig. 2a, inset graph, lower right corner, HE_11_ mode) and by the dielectric multilayers (Fig. 2a, inset graph, top left corner, BSW-1D mode). For example, their effective indexes are much closer and increase with increasing radii of the nanofibres, *R*. The electric fields of these two modes are both attached around the surface of the nanofibre (inset graphs in Fig. 2b, *R*=125 nm). To quantitatively present the field distribution, intensity profiles along the lines passing through the centre of nanofibre are plotted in Fig. 2b (corresponding to line parallel to the *X* axis, *Y*=−62.5 nm) and Fig. 2c (line parallel to the *Y* axis, *X*=0 nm), which show nearly identical features for the two modes in the case of peak location and decay path in air. Within the substrates the fields are different. The propagating mode consists in the combination of a 2D planar waveguide and dielectric multilayer (which together forms a new structure that supports a BSW mode). The mode propagating along the nanofibre on the dielectric multilayer substrate is similar to the mode of a nanofibre in air (HE_11_ mode, fundamental mode of a symmetric nanofibre), so it can similarly be characterized as a BSW. In this case, the BSW field is not attached to a 2D material but is confined on the surface of a 1D material and, in this case, we call it a BSW-1D mode.

**Figure 3:**
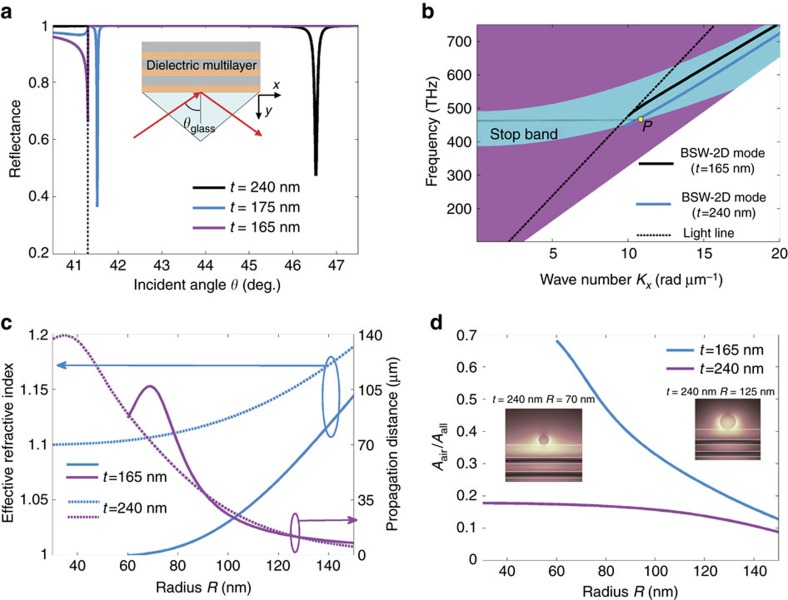
Numerical comparisons of DLBSW and BSW-1D modes. (**a**) Calculated reflectance of the dielectric multilayer versus incident angle under TE illumination for different thicknesses *t* of the top SiO_2_ layer. The dotted line denotes the critical angle between the glass substrate and air. (**b**) The TE waves band structure for the dielectric multilayer. The turquoise zone is the stop band. The dispersion relations for BSW-2D modes with *t*=165 nm (magenta line) and *t*=240 nm (blue line) are also demonstrated. The dashed line denotes the light line in air. The point *P* is corresponding to the excitation wavelength 633 nm. (**c**) The effective refractive indexes and the propagation distance of the BSW-1D (*t*=165 nm) and DLBSW (*t*=240 nm) modes as a function of the nanofibre radius; (**d**) the ratio of mode energy (*A*_air_/*A*_all_) for DLBSW and BSW-1D modes versus the nanofibre radius. The insets show the electric field distributions for different nanofibre radius (*R*=70 nm and *R*=125 nm) with *t*=240 nm.

As described in the literature, a BSW can also be guided along a polymeric stripe (termed DLBSW) where the dielectric multilayers themselves support the BSW-2D mode^24^. If we set the thickness of the top SiO_2_ layer, *t*, to be 240 nm, we can detect the excitation of the BSW-2D mode from the narrow dip at 46.53° by using the calculated angular dependence reflectance curves of the multilayers (Fig. 3a). When *t* ≤165 nm, as discussed above, there was no dip for resonant angles larger than the critical angle (indicated by the black dotted line), meaning there was no excitation of BSWs. The smaller dip, which is much closer to and smaller than the critical angle, is caused by transmission into air. However, when *t* is increased a little, such as for *t*=175 nm, BSWs appear with a resonant angle of 41.52°. Therefore, *t*=165 nm is close to the cutoff thickness for the generation of a BSW-2D mode on a multilayer. The TE wave band structure for the dielectric multilayer is also shown in Fig. 3b. The dispersion relations for BSW modes with *t*=165 nm and *t*=240 nm are also demonstrated. The point *P* shown in Fig. 3b is corresponding to the excitation wavelength (633 nm). It is noted that the dispersion curve for BSW mode with *t*=165 nm approaches to the light line in the air around the excitation wavelength. It means that the BSW mode is nearly cut off for *t*=165 nm. For the BSW mode with *t*=240 nm, the wave number *K*_*x*_ of BSW mode at the excitation wavelength is outside of light line in air and can be excited with the Kretschmann configuration as shown in Fig. 3a.

For comparison, when a nanofibre is placed on a multilayer with *t*=240 nm, its effective index is larger than that of a nanofibre in air or on a multilayer with *t*=165 nm (Fig. 3b). The propagation lengths of the BSWs inside the nanofibre (*R*=125 nm) with *t*=165 nm and *t*=240 nm are also plotted (Fig. 3c); they show a similar dependency on *R*. However, the most obvious difference between the two cases is in the electric field distribution. When the nanofibre (*R*=125 nm) is placed on a multilayer with *t*=240 nm, the field shifts a little into the top SiO_2_ layer (inset graph of Fig. 3d, right corner). If *R* is decreased to 70 nm, the spread of the optical field into the top SiO_2_ layer increases significantly (left corner Fig. 3d) and will gradually evolve into a BSW-2D mode (such as for *R*=40 nm, Supplementary Fig. 2a). For the case of a nanofibre on a multilayer (*t*=165 nm), the optical field does not spread into the multilayer even with decreasing radius (such as for *R*=70 nm, Supplementary Fig. 2b). The spread of the electric field into the multilayer can be attributed to the mode hybridization between nanofibre mode and BSW-2D mode. The mode hybridization phenomenon is discussed in Supplementary Figs 3 and 4.

To further visualize the difference between the BSW-1D and DLBSW modes, the ratio of the mode energy localization in air (*A*_air_) to the total mode energy (*A*_all_) was calculated and is shown in Fig. 3d (ref. 30). *A*_air_ and *A*_all_ are defined as follows:


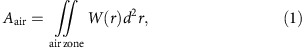



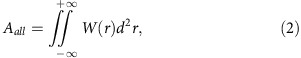


where *W*(*r*) is the electromagnetic energy density, which is given by:


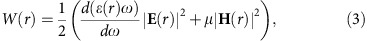


where 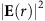 and 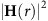 are the intensity of the electric and magnetic fields, respectively, and *ɛ*(*r*) and *μ*_0_ are the electric and vacuum magnetic permittivity respectively. *r* represents a spatial position (*r*=*r* (*x*, *y*, *z*)). The ratio of *A*_air_/*A*_all_ versus radius *R* is plotted in Fig. 3d demonstrates that the optical field of the BSW-1D mode is more confined in air than that of the DLBSW mode, especially for smaller *R*. This observation is consistent with the electric field distribution shown in the insets of Figs 2d and 3d, where most of the DLBSW field diffuses into the multilayer for small *R* but that of the BSW-1D mode does not. The electric field distribution of the BSW-1D mode is more favourable for sensing applications than that of the DLBSW, as the data in Supplementary Fig. 2c demonstrates.

The similarities between the BSW-2D and TE_0_ mode, and the BSW-1D and HE_11_ mode show that the essence of a BSW may be ascribed to these fundamental modes in a planar waveguide or a 1D nanofibre. Owing to the PBG of the multilayers, the optical field of the modes cannot leak into the substrate and instead retains a similar distribution as in air. However, if a planar waveguide or nanofibre with very small dimensions, such as *d*=100 nm or *R*=125 nm, is placed on a bare glass substrate, no mode is observed. This result is caused by leakage radiation consisting entirely of optical energy into the glass substrate. This problem is theoretical solved if the bare glass substrate is replaced with a dielectric multilayer containing a PBG, as discussed above.

### Experimental demonstration of DLBSW and BSW-1D

Multilayers of the same structural parameters as in Fig. 1a were fabricated via plasma-enhanced chemical vapour deposition (PECVD) and characterized with a scanning electron microscope, as shown in Supplementary Fig. 5. The top SiO_2_ layer thickness was about 165 nm or 240 nm. We named these two samples ‘Multilayer-165' and ‘Multilayer-240', respectively. A single Nylon 6 nanofibre with a radius of ∼125 nm (similar to the one shown in Fig. 2b) was made by electrospinning and then transferred onto the two multilayers (Supplementary Fig. 5). Nylon-6 (also known as polyamide 6) possesses excellent physical and mechanical properties, and is one of the most widely used synthetic polymer fibres^31^. A decisive advantage of polymer nanofibres compared with metallic nanowires or structures made by electron beam lithography (EBL), is that a fibre can be easily functionalized to provide thermo-optical, electro-optical or all-optical functionalities, which can be used for the development of active nanophotonic components^28^. In our experiment, the nanofibre was doped with dye molecules (Nile Red, NR) so it could be employed as an active element. The doping process is convenient, because the NR molecules act as point sources distributed in the nanofibre and their emission will couple into the optical modes available in the nanofibre/multilayer structure (Fig. 2a). The fluorescence from NR can be excited using far-field illumination, which allows the optical properties of these modes to be characterized via fluorescence images.

### Coupling of fluorescence with a BSW-1D

A tightly focused laser beam with a wavelength of 532 nm is focused by an oil-immersed objective (× 60, numerical aperture (NA) 1.49) to excite the NR molecules inside the nanofibre. Front focal plane (FFP) and back focal plane (BFP) images of the fluorescence from the nanofibre were measured with two detectors (cameras). The detailed experimental setup of the home-built leakage radiation microscope is presented in Supplementary Fig. 5 and described in the Methods section. Emission from the fibre was selected using a band pass filter (full width at half maximum (FWHM) of 3.0±0.6 nm, centre wavelength of 632.8±0.6 nm, Thorlabs Inc., USA) placed before the cameras.

**Figure 4:**
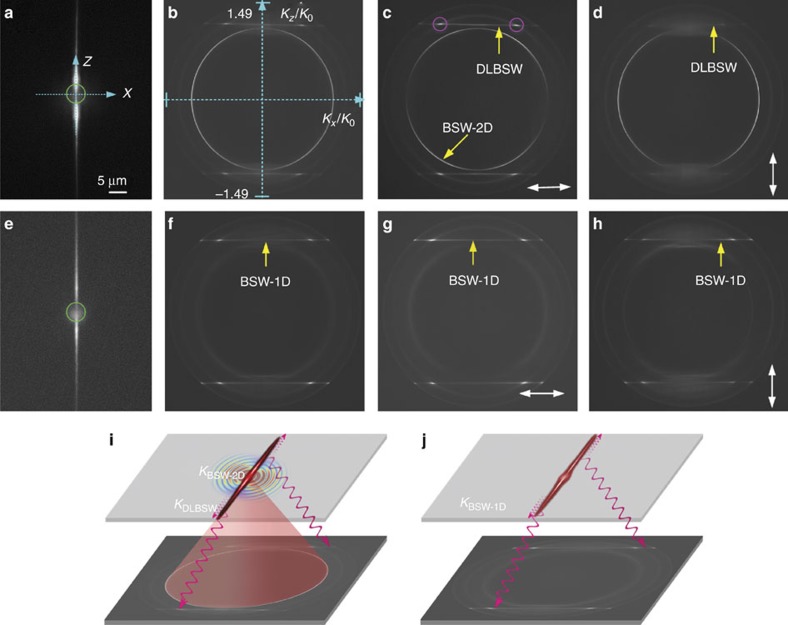
Experimental comparisons of BSW-1D and DLBSW modes. FFP fluorescence image of nanofibre on the Multilayer-240 sample (**a**) and Multilayer-165 sample (**e**). The radius of the nanofibre, *R*, was ∼125 nm. The green circles indicate the focal spot of the laser beam. The *Z* axis is defined as along the fibre and the *X* axis is perpendicular to the nanofibre (in the plane of the multilayer); **b**–**d** and **f**–**h** show the corresponding BFP images of **a** and **e**, respectively; **c**,**d** and **g**,**h** show the BFP images with a polarizer before the detector, the double arrow-headed white lines indicate the orientation of the polarizer. (*K*_*x,z*_*/K*_0_)_max_=1.49 is given by the NA of the objective. The two magenta circles in **c** indicate the cross points between the bright line and one of the weak rings. (**i**,**j**) The corresponding schematic diagrams of **a**,**b** and **e**,**f**, respectively. The top plane of the diagram represents the FFP image and the bottom the BFP image. The fluorescence wavelength was selected by the bandpass filter as 632.8 nm.

The FFP fluorescence images of single nanofibre on the Multilayer-165 and Multilayers-240 samples are shown in Fig. 4a,e, respectively. At the laser focus (marked with green circles), the fluorescence is bleached in the middle of the fibre owing to the intense excitation field. The BFP images with (Fig. 4c,d,g,h) and without (Fig. 4b,f) a polarizer before the camera reveal a dramatic difference between the two samples. The BFP image for Multilayer-240 (Fig. 4b–d) displays a bright, thin ring of emission—this ring is absent from the BFP image for Multilayer-165 (Fig. 4f–h).

The bright ring (Fig. 4b) is the well-known BSW-coupled emission, meaning that the excited BSW propagates in all azimuthal directions (from 0° to 360°) on the top surface of the multilayer^32^. This kind of surface wave can be viewed as a BSW-2D mode, which is the medium for 2D optics similar to how an SPP is for a metallic film. From the known NA of the objective (1.49) and the diameter of the bright ring, the effective refractive index (*K/K*_0_) of the BSW-2D mode can be derived as 1.10, where *K*_0_ is the free-space wavevector. The corresponding resonant angle *θ* (n*sin* θ*=*K/K*_0_, *n* is the refractive index of the glass substrate) is ∼46.45°, which is consistent with the calculated value in Fig. 3a. From the known orientation of the polarizer, we can judge that the ring has azimuthal polarization, meaning every spot on the ring is polarized in the azimuthal direction, which corresponds to TE or *s*-polarization.

An interesting and important feature in the BFP images are the bright horizontal lines at the constant *K*_z_*/K*_0_ (effective refractive index) value of ±1.15 (Fig. 4b) or ±1.08 (Fig. 4f), which is a typical signature of propagating waves inside a single, straight nanofibre (along the *Z* axis (Fig. 4a), ±*Z* directions) or of 1D waveguides^33^,^34^,^35^,^36^. However, when the same nanofibre was placed on a glass substrate, we cannot observe this bright horizontal line, so this propagation mode is associated with BSWs. In the BFP images, the appearance of the bright ring means that the BSW propagating inside the nanofibre is a DLBSW mode (Fig. 4b–d); without the ring we have a BSW-1D mode. The simultaneous appearance of both rings and lines means that the energy from the NR molecules not only couples into the BSW and propagates along the nanofibre but also that the energy disperses on the top surface of the multilayer. Here, the ring is even much brighter than the lines, meaning that most of the fluorescence energy are dispersed on the surface but not confined into the nanofibre. The effective refractive indexes (*K*_z_*/K*_0_), also called the real parts of the propagation constants for a DLBSW or BSW-1D mode, are also consistent with the calculated values shown in Fig. 2a. Conversely, as for a SPP on a 2D metal film, a BSW-2D mode is also sensitive to the incident wavelength. For example, for the same multilayer, although the BSW-2D mode cannot be excited at longer wavelengths, such as at 640 nm, it can be excited at shorter wavelengths, such as at 600 or 620 nm (Supplementary Fig. 6). If a nanofibre was placed on this multilayer, the BSW-1D mode would be transformed to a DLBSW mode for decreasing incident wavelengths, as in Supplementary Fig. 6. The fluorescence BFP images can also be numerically simulated with the finite-difference time-domain method (Supplementary Note 1), as shown in Supplementary Figs 7 and 8 (refs 37, 38), which are consistent with the experimental results. Both simulated and experimental BFP images give the difference between the BSW-1D mode and the DLBSW mode. In contrast to the structure for DLBSWs, where BSW-2D modes exist simultaneously, the structure for the BSW-1D mode only sustains BSWs adhering to a nanofibre (no bright ring appear on the BFP images), which can greatly suppress the background noise and force the BSWs to propagate only along a 1D material. In this way, the optical energy of a BSW will be mainly confined around the nanofibre and is not dispersed on other surface of the multilayer, which would lead to a high-sensitivity efficient transport of the optical energy to surrounding medium.

The schematic diagrams in Fig. 4i,j illustrates the formation of the thin ring and horizontal lines, and also present the correlations between the FFP and BFP images. For the nanofibre on Multilayer-240, the BFP image (Fig. 4b) displays a bright ring; therefore, we know that there are surface waves propagating on the multilayer, as illustrated in Fig. 4i (top plane). These surface waves are not as clearly visible in the FFP image (Fig. 4a), because their intensity is masked by the fluorescence from the fibre itself. The surface waves (BSW-2D) show leakage radiation into the glass substrate and form the thin ring (Fig. 4b). In contrast, the schematic in Fig. 4j shows that coupled emission from the BSW-1D structure results in straight line perpendicular to the wire. There is no bright thin ring in Fig. 4f–h, which indicates that the Multilayer-165 sample cannot sustain a BSW-2D mode at a wavelength of 632.8 nm. The horizontal lines with constant *K*_*z*_/*K*_0_ signify that the wave vector (*Z*-component, *K*_*z*_) of the propagating wave in the nanofibre is constant. There are two horizontal lines on a BFP image, which means that the waves propagate in both the +*Z* and −*Z* direction. The horizontal line fills the detection window along the *K*_*x*_ axis, which means that the *K*_*x*_/*K*_0_ (*X*-component of the wave vector) value has a wide range. The mechanism behind this phenomenon can be explained as follows: as we know, the BFP image is a Fourier transform of the FFP image. The formation of the horizontal line is merely a property of the Fourier transform of optical waves confined by a 1D structure. For example, the Fourier transform of a Dirac pulse (infinitely small range of *x*) is a constant (meaning infinitely large range of the reciprocal parameter, *K*_*x*_). Similarly, a laterally confined optical field (that is, finite, small range of *x*) would give rise to a large spread of wave vector values. The smaller the spatial range of *x*, the greater the range of *K*_*x*_. In our experiment, we see the optical field confined by the nanofibre whose lateral dimension (along the *X* axis, Fig. 4e) is very small (radius *R*=125 nm, small range of *x*); therefore, its Fourier transform gives rise to a large spread of *K*_*x*_ (*X*-component wave vector).

It should be noted, except for the bright ring associated with BSW-2D mode (Fig. 4b–d), there are other thicker, but weaker, rings that appear in the BFP images (Fig. 4b–d, f–h and Supplementary Fig. 6e). These rings are associated with the multilayer but not the nanofibre and their electric field distributions are mainly confined inside the multilayer rather than the top surface; hence, they will not mix with the BSWs in optical signal transmissions^35^. There are two bright spots (marked with magenta circles, Fig. 4c) on each bright line, which are the results of the coherent superposition between leaky fluorescence coupled into the BSW and into the internal modes (Supplementary Fig. 6e) of the multilayer. Their superposition means that they have the same leakage radiation angles into the glass substrate but do not overlap in the optical field distribution. These weaker rings (associated with internal modes of the multilayers) could not be clearly resolved in BFP images in previous publications^36^, where a long-pass filter, but not a narrow bandpass filter, for fluorescence was used (Supplementary Fig. 6f); here, they could be resolved and their wavelength dependence can be observed.

### Excitation of BSW-1D with laser beam

**Figure 5:**
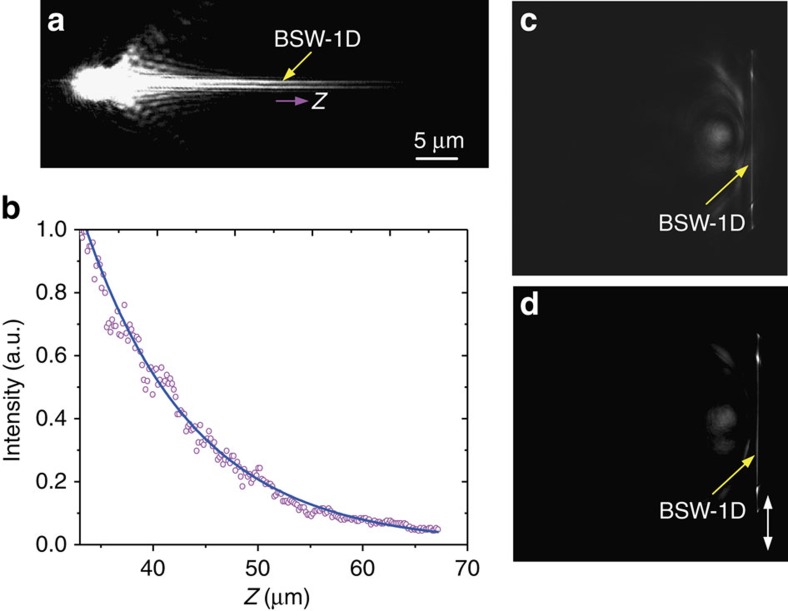
BSW-1D excited by laser beam from fibre tip. (**a**) FFP image of the laser beam propagating along the nanofibre. The laser beam has a wavelength of 632.8 nm. (**b**) Intensity distribution along the nanofibre (*Z* direction, in **a**) is shown (magenta dots). The blue solid line is an exponential fit to the data and was used to extract the propagation distance of the BSW-1D. (**c**,**d**) The corresponding BFP image with and without a polarizer before the detector. The double arrow-headed white line in **d** shows the polarizer orientation. The polarizer can erase optical noise around the bright line, making the line more distinct.

We will demonstrate that the laser beam from the fibre taper can also be directly coupled into the polymer nanofibre and form a BSW-1D mode, as shown in Fig. 5. The laser beam with a wavelength of 632.8 nm was coupled into a single mode silica fibre with a nano-taper, which is close to the boundary of the polymer nanofibre (Supplementary Fig. 5). The radius of the nanofibre was about 125 nm. Apart from the bandpass filter, which had a central wavelength of 632.8±0.6 nm, no other filters were used. The FFP image (Fig. 5a) shows that the light propagates along the nanofibre, whereas the optical field is confined to the surface of the fibre (but not inside it), which is consistent with a numerical simulation shown in Fig. 2b. This effect is suitable for optical transport or sensing applications. Importantly, the BSW's location differs between the 2D plane and in 1D materials. The BFP image (Fig. 5c,d) shows a brilliant line but no bright ring, which verifies the excitation of the BSW-1D but not the DLBSW mode. In contrast to the excitation of BSWs with fluorescence, where there are the two bright lines and other rings (Fig. 4), there is only one bright line here, meaning the BSWs mainly propagate along one direction. This phenomenon demonstrates that with the aid of an optical fibre taper only the BSW-1D, and no other modes, can be excited efficiently. The propagation length of the BSW-1D mode can be derived from the FFP image (Fig. 5b). The intensity distribution along the nanofibre (red dots, *Z* direction) is accurately described by an exponential curve (blue solid line) from which the propagation distance can be inferred as 10.42 μm. The experimental value is slightly smaller than the calculated propagation distance (12.21 μm in Fig. 3c), which is caused by the scattering loss, owing to the roughness of the surface of the polymeric nanofibre. The propagation length for DLBSW mode on the structure Multilayer-240 was also measured (Supplementary Fig. 9). The length is about 9 μm and nearly the same as the BSW-1D, which is consistent with the calculations in Fig. 3c. In this case, the BSW-2D mode is excited simultaneously by the laser beam as represented by the bright arcs (Supplementary Fig. 9c,d).

### Functional fluorescent nanofibre

**Figure 6:**
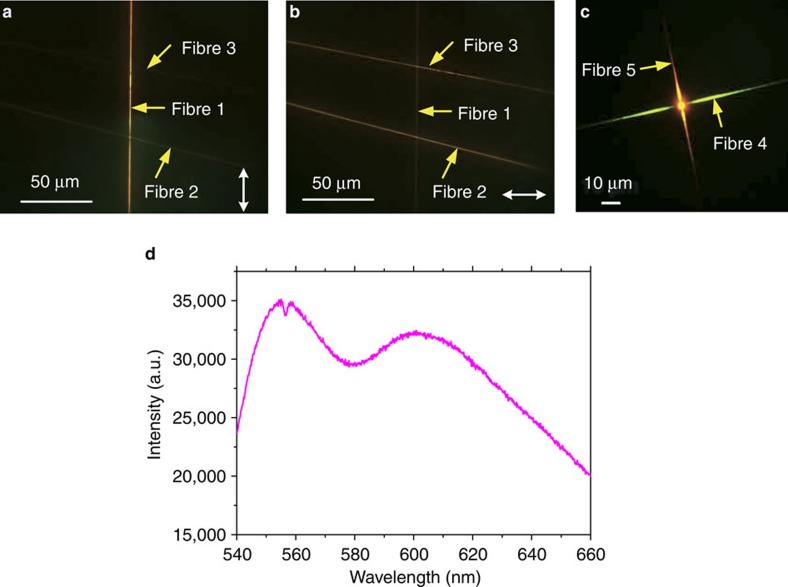
Functional fluorescent nanofibre. (**a**) Fluorescence image of three cross nanofibres on the multilayer. The polarization direction of the excitation laser beam is vertical (**a**) or horizontal (**b**). The double headed arrows on **a** and **b** indicate the polarization direction of the laser beam. The laser beam are expanded and illuminated the sample from the top side (Supplementary Fig. 5). (**c**) Fluorescence images of two cross-nanofibres doped with Rh6G (Fibre 4) and NR (Fibre 5) molecules, respectively. The excitation laser beam is focused onto the cross-point. A colour charge-coupled device detector combined with a long pass filter was used. (**d**) Corresponding fluorescence spectrum taken by a spectrometer.

One advantage of polymeric nanofibre over that of metallic structure is the easy doping with functional units. For structures doped with fluorescent molecules, such as dielectric nano-stripes, made using optical lithography or EBL, the molecules always have random orientations. In our experiment, although the NR molecules were dissolved in the formic solution and initially have random orientations, they can have an ordered arrangement after electrospinning process, which is verified by following fluorescence images (Fig. 6). The forming principle of the ordered dye molecules can be ascribed to the electron-spinning procedure where a high-voltage electrostatic field was used. The electrostatic field can make the polar molecule (NR) orderly arranged during the formation of nanofibre. Three cross-nanofibres on the dielectric multilayer were uniformly illuminated with a collimated and linearly polarized laser beam of 532 nm wavelength from the top (Supplementary Fig. 5). For Fibre 1, its long axis is parallel to the polarization direction of the laser beam, whose fluorescence intensity (Fig. 6a) is much stronger than that from Fibre 2 and Fibre 3. When the polarization direction is changed to be horizontal (Fig. 6b), the fluorescence from Fibre 1 is significantly reduced and the fluorescence from Fibre 2 and Fibre 3 becomes stronger than that from Fibre 1. This phenomenon clearly shows that the orientation of the NR molecules is along the long axis of the nanofibre^39^. Polarization sensitivity of the fluorescent nanofibre to external excitation beam provides a new means to actively control the BSW mode, which cannot be realized with the nanowire made with lithography method or other methods mentioned with random molecular orientations.

The use of electrospun nanofibre can have additional advantages over polymeric nano-stripes made by optical beam lithography or EBL. When fabricating such stripes for SPPs or BSWs, the active molecules (such as dyes) are always dissolved in liquid solution, which is then spin-coated onto a substrate. After the exposure, developing and fixing processes, the active molecules doped dielectric nanofibres can be obtained on the substrate. It is difficult to obtain waveguides with different kinds of doping molecules on the same substrate by using lithography. For example, to the best of our knowledge, there are numerous reports on active dielectric-loaded SPP waveguides or DLBSWs but we have found no reports on two or three nano-stripes doped with different molecules on the same substrate. In our work, electron spinning was used to make the nanofibres doped with different molecules and then placed on the same substrate (Fig. 6c). A colour type charge-coupled device detector combined with a long-pass filter (cutoff wavelength at 550 nm) was used to obtain the image. The green (Fibre 4, doped with Rhodamine 6G (Rh6G)) and red (Fibre 5, doped with NR) nanofibres appear simultaneously on the multilayer. The laser beam with a wavelength at 532 nm is focused onto the cross-point of the two fibres. The corresponding fluorescence spectrum is shown in Fig. 6d, which gives out two peaks. Colourful BSWs propagating inside the nanofibres are obtained (Fig. 6c). The combination of electron spinning nanowires and dielectric multilayer provides a new method to realize BSWs (both DLBSW and BSW-1D) working at different wavelengths on the same substrate.

## Discussion

The present research demonstrates the identification of a type of BSW, enabling the optical signals' transportation along single polymeric nanofibre of small radius on a solid substrate. This mode extends the concept of BSWs from 2D to 1D, which had not been proposed before and is helpful for conceptualizing BSWs. The advances from the commonly investigated BSW-2D to the novel BSW-1D mode means that BSWs can be more favourable for transmitting optical signals, data processing and optical sensing. To the best of our knowledge, this is the first reported use of electrospun polymer nanofibres of very small radius to guide BSWs, which will establish a bridge with the surface waves and various functional nanowires. This can lead to a substantial range of new nanometric scale devices. There is a great deal of flexibility in our design because of the easy doping of the materials and the wide range of plastic fibres that can be used. For example, the polymer nanofibre might be replaced by any biochemically functional material suitable for molecular recognition (such as recognition of the DNA with long chain). The proposed structure for the BSW-1D mode can provide disruptive opportunities in waveguide-based biosensing schemes^40^,^41^.

The materials of the multilayers and nanofibre for the BSW-1D mode also can be replaced with many other dielectrics to allow for applications over a wide range of wavelengths. Materials such as silicon can be used, which is important, as silicon is widely used in integrated circuits and in dielectric metasurfaces. Therefore, the BSW-1D mode may become important in this exciting, novel research field where it could have the same function that SPPs currently have in metasurfaces. We believe our work may open up new opportunities in the field of nanophotonics.

## Methods

### Experimental setup of leakage radiation microscopy

The detailed experimental setup for characterizing BSWs is shown in Supplementary Fig. 5. The incident laser beam (532 nm wavelength) is first expanded by a lens array to fill the rear aperture of an oil-immersed objective (× 60, NA, 1.49). The incident beam is focused onto the middle of the nanofibre positioned on the multilayer surface. The excited fluorescence will then be collected by the same objective. A 550 nm long-pass filter or a narrow bandpass filter (FWHM of 3±0.6 nm, centre wavelength of 632.8±0.6 nm, Thorlabs Inc.) is used to reject the incident laser beam and to allow the fluorescence at the selected wavelength to reach the collection lens. Another series of bandpass filters with a FWHM of 10±2nm and centre wavelengths of 610±2, 620±2, 630±2 and 640±2 nm are used to investigate the sensitivity of BSWs to changes in wavelength. A beam splitter is used to split the fluorescence into two beams. The first beam is used for FFP imaging (DS-2MBWc Camera, Nikon, Japan) and the second one is for BFP imaging (Neo-sCMOS, Andor, UK). There is an additional laser with a wavelength of 632.8 nm that couples into a silica fibre with a nano-taper at one end. The fibre taper is used to couple the laser beam into the polymeric nanofibre (Fig. 5). An additional laser at 532 nm wavelength on the top side of the objective, which passes through a single-mode fibre and a collimator to evenly illuminate the ultra-long fluorescent nanofibre. A polarizer and a half wave-plate were used to tune the polarization direction of the laser beam to investigate the polarization sensitivity of the nanofibre (Fig. 6). The three lasers were not turned on simultaneously. The camera for the FFP image can be replaced with a spectrometer (ihR 550, HORIBA Scientific) to measure the fluorescence spectrum (Fig. 6d).

### Fabrication of all-dielectric multilayers

The dielectric multilayers were fabricated via PECVD (Oxford System 100) of SiO_2_ and Si_3_N_4_ on a standard microscope cover glass (0.17 mm thickness) at a vacuum <0.1 mtorr and temperature of 300 °C. Before the PECVD of a dielectric multilayer, the cover glass was cleaned with piranha solution and then with nanopure deionized water and dried with an N_2_ stream. SiO_2_ is the low (L) refractive index dielectric and Si_3_N_4_ is the high (H) refractive index dielectric. Their thicknesses are 105 and 88 nm, respectively. There are 14 layers in total. The thickness of the top SiO_2_ layer was ∼165, 240 or 167 for the Multilayer-240 sample (Fig.4a–d and Supplementary Fig. 9), Multilayer_-_165 sample (Figs 4e–h and 5) or Multilayer-167 sample (Supplementary Fig. 6), respectively.

### Fabrication of polymer nanofibre

The typical procedure for the fabrication of fluorescent electrospun Nylon nanofibres is given below. A 2 ml formic solution (solvent for the Nylon) containing 0.6 g Nylon 6 and 6 mg of NR was ejected at a continuous rate using a syringe pump through a stainless steel needle. For the preparation of Rhodamine 6G (Rh6G) embedded Nylon nanofibre, 2 ml formic acid solution containing 10 mg of R6G and 0.6 g of Nylon was used. A voltage of 10 kV was applied to the needle with a high voltage power supply and a feed rate of 0.1 mm min^−1^ was maintained with a syringe pump. A collector (the glass substrate with the fabricated dielectric multilayer) was placed at a distance of 10 cm from the needle tip to collect the nanofibre.

### Data availability

The data that support the findings of this study are available from the corresponding authors upon reasonable request.

## Additional information

**How to cite this article:** Wang, R. *et al*. Bloch surface waves confined in one dimension with a single polymeric nanofibre. *Nat. Commun.* **8,** 14330 doi: 10.1038/ncomms14330 (2017).

**Publisher's note:** Springer Nature remains neutral with regard to jurisdictional claims in published maps and institutional affiliations.

## Supplementary Material

Supplementary Information Supplementary Figures and Supplementary Note
