# Supplementary material for: Bloch surface waves confined in one dimension with a single polymeric nanofibre
Source: Nat Commun. 2017 Feb 3;8:14330. doi: 10.1038/ncomms14330 (PMC5296743; doi:10.1038/ncomms14330)
Supplement: Supplementary Information — Supplementary Figures and Supplementary Note [file ncomms14330-s1.pdf]

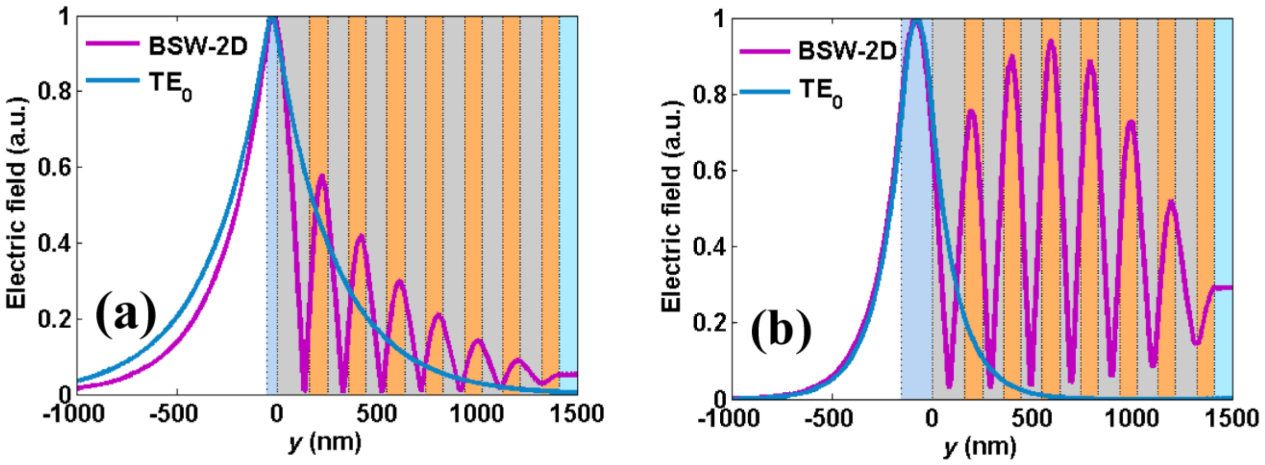

Supplementary Figure 1 | Electric field distributions for BSW-2D and TE_0 modes with different waveguide thicknesses. (a) Waveguide thickness *d* = 50 nm; (b) waveguide thickness *d* = 150 nm. The thickness of the top SiO_2 layer was 165 nm. Y = 0 nm indicates the interface between the multilayer and the planar waveguide (Fig. 1a).

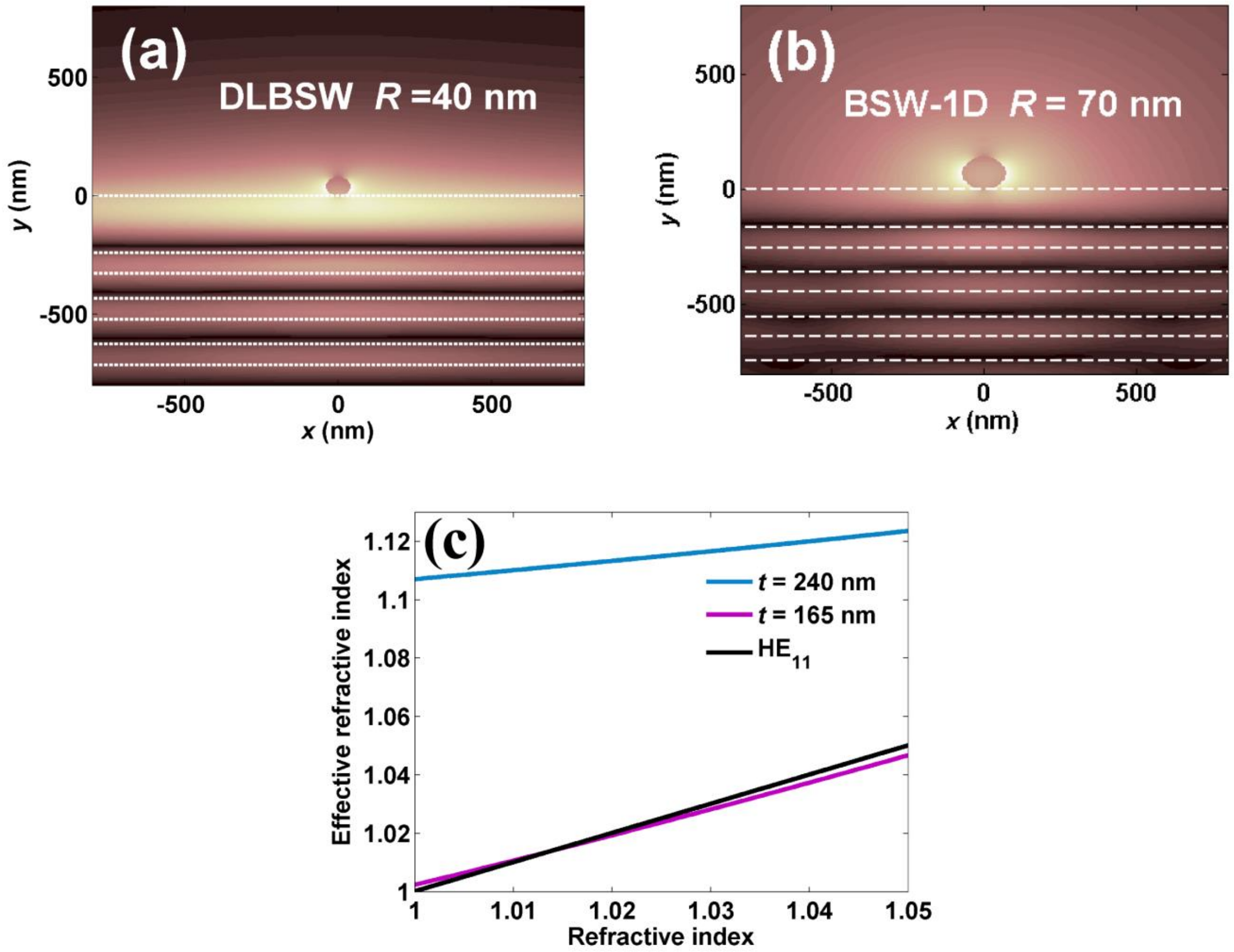

Supplementary Figure 2 | Electric field distribution of DLBSW and BSW-1D modes, and their sensing ability. (a) The electric field distribution for the DLBSW mode with the geometric parameters t = 240 nm and R = 40 nm; (b) the electric field distribution for the BSW-1D mode with the geometric parameters t = 165 nm and R = 70 nm; (c) the effective refractive indexes of BSW modes with different thicknesses t and HE_{11} mode of a nanofibre as a function of the surrounding refractive index. The radius of the nanofibre was 70 nm. The larger slope of the curves for t = 165 nm (slope = 0.9) and HE_{11} mode (slope = 1) compared with for t = 240 nm (slope = 0.33) indicates a higher sensitivity to the surrounding medium.

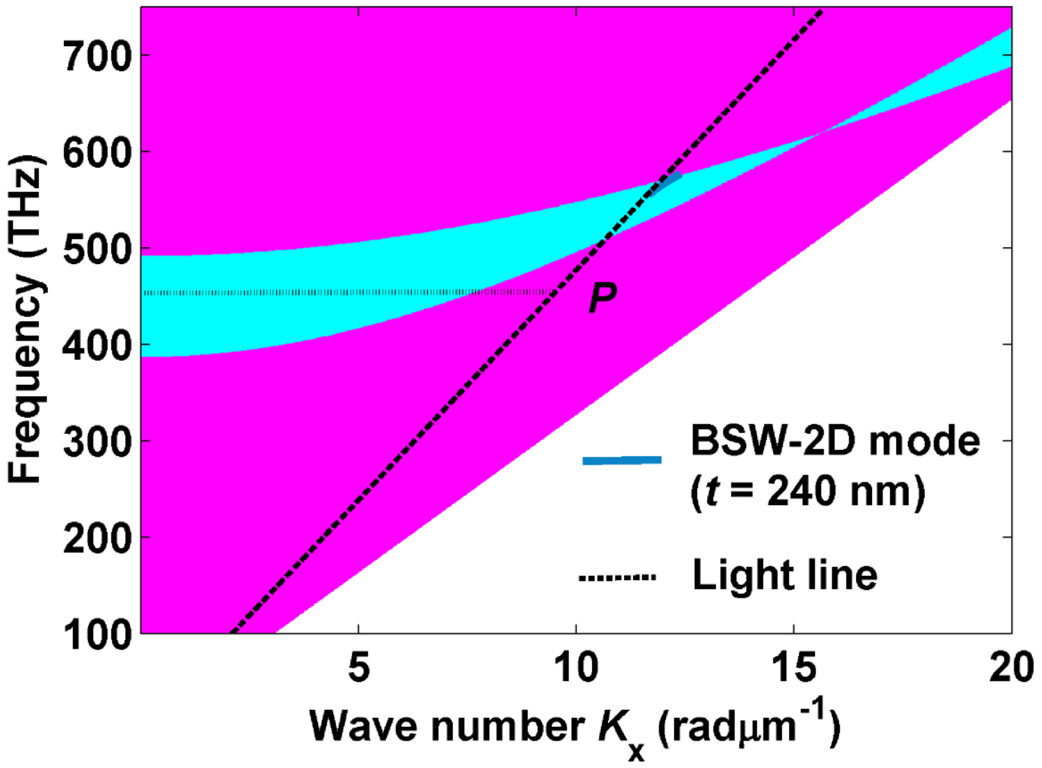

Supplementary Figure 3| The TM waves band structure for the dielectric multilayer. The point P is corresponding to the excitation wavelength. It is noted that the cut off wavelength for BSW-2D mode with TM polarization is much smaller than the excitation wavelength, as the thickness of top layer is 240 nm. Therefore, only TE BSW-2D mode can be excited in the paper.

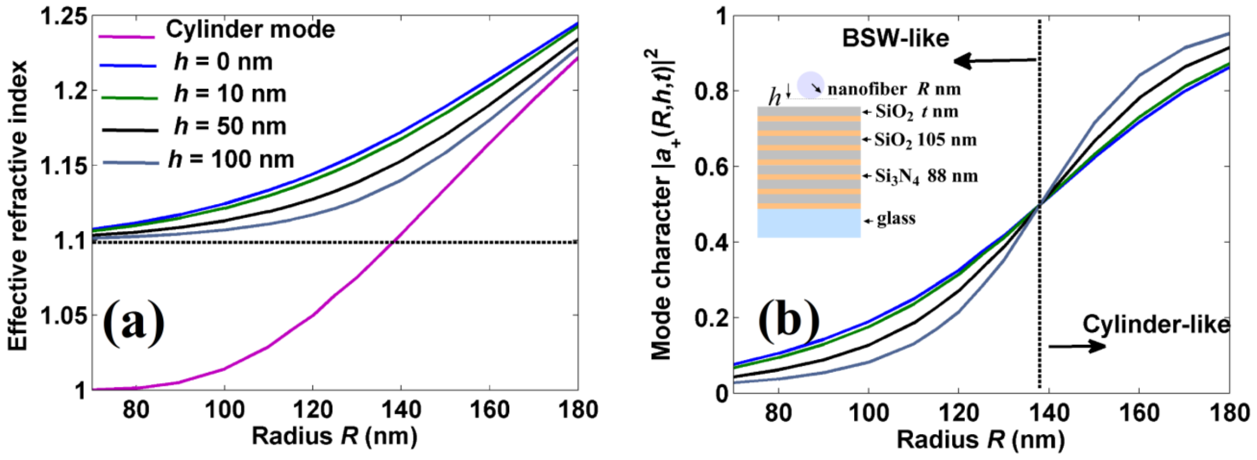

Supplementary Figure 4| The hybridization of the nanofibre and BSW-2D modes as modelled by coupled mode theory. (a) The effective refractive index of hybrid modes for a range of gap width h and the nanofibre radius *R*. The dashed line denotes the pure BSW-2D mode. (b) The mode character determined by Eq. (S2). Inset in (b) shows the schematic structure. A nanofibre with radius *R* is separated from the dielectric multilayer by a nanoscale gap of width h. The thickness *t* is 240 nm. The TE BSW-2D mode can be guided by the structure in this geometrical parameter. The mode hybridization will be happened, as the effective refractive indices of BSW-2D and nanofibre modes are overlapped with the decreasing of the nanofibre radius. The coupled mode theory in Ref. [32] is used to study the mode hybridization behavior. The two possible hybrid modes can be expressed as the superposition of the pure BSW mode and the HE_{11} mode of nanofibre.

$$\psi_{\pm}(R,t,h) = a_{\pm}(R,t,h)\psi_{cyl}(R) + b_{\pm}(R,t,h)\psi_{BSW}(t) \quad \text{(S1)}$$

Similar to the process outlined in Ref. [32], the square norm of the cylinder mode amplitude $|a_{+}(R,t,h)|^2$ can be expressed as:

$$|a_{+}(R,t,h)|^2 = \frac{n_{hyd}(R,t,h) - n_{BSW}(t)}{((n_{hyd}(R,t,h) - n_{cyl}(t)) + (n_{hyd}(R,t,h) - n_{BSW}(t)))} \quad \text{(S2)}$$

However, the TE BSW mode is cut off as the thickness *t* decreases to 165 nm. Then, the hybridization between fibre mode and BSW-2D mode is not existed in the structure.

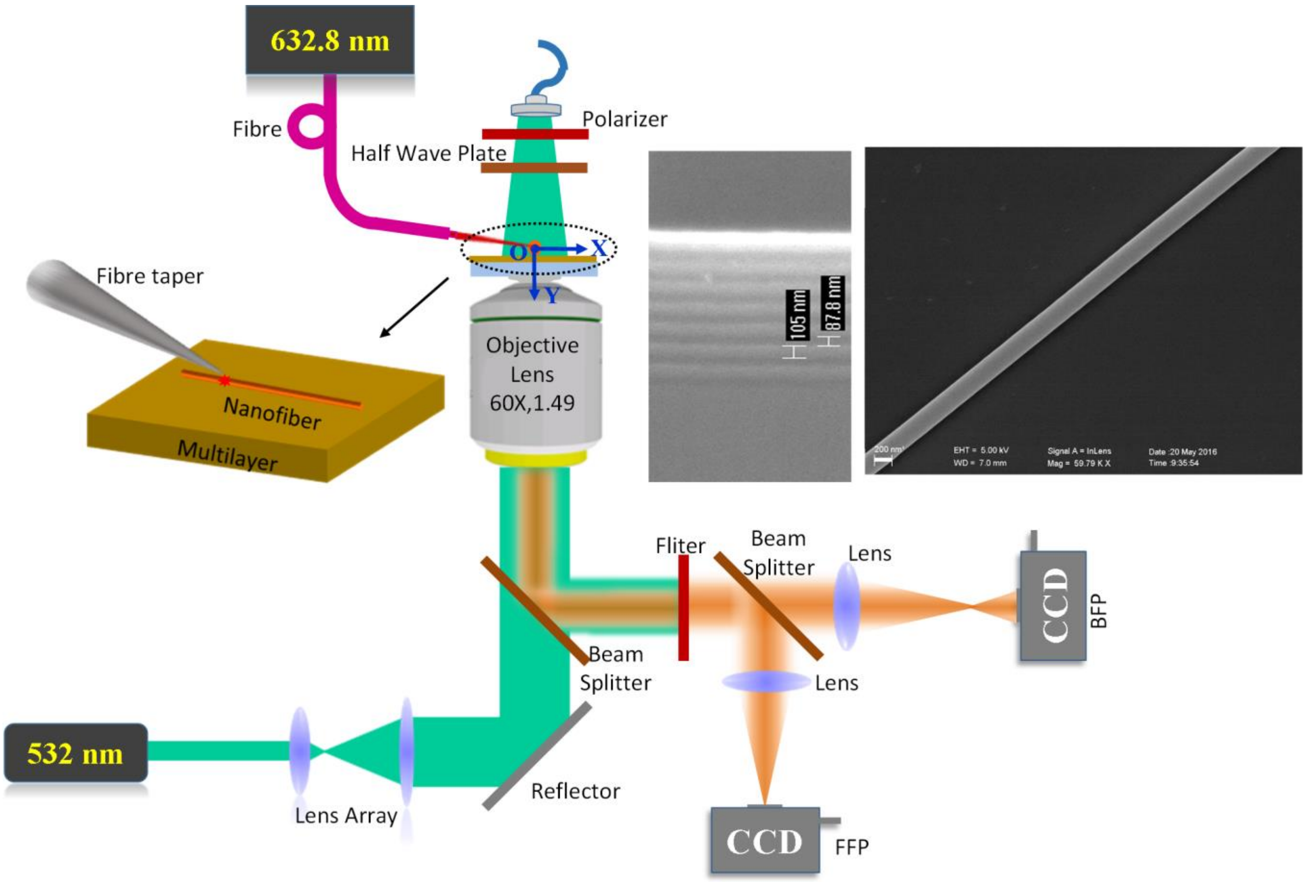

Supplementary Figure 5 | Schematic diagram of the optical setup for leakage radiation microscopy. The back focal plane (or named as Fourier plane image) and front focal plane (or named as Direct-space image) of the objective can be imaged onto two cameras. A laser with a wavelength of 532 nm was used to excite the fluorescence (for Fig. 4, Fig. 6c, 6d, Supplementary Figure 6). A laser beam with a wavelength of 632.8 nm was couple into the nanofibre with the aid of a fibre taper (for Fig. 5, Supplementary Figure 9). There is additional laser at 532 nm wavelength on the top side of the objective, which passes through a single mode fibre and a collimator to evenly illuminate the ultra-long fluorescent nanofibre. A polarizer and a half wave-plate were used to tune the polarization direction of this laser beam to investigate the sensitivity of the nanofibre to the incident polarization (for Fig. 6a, 6b). The three lasers were switched on or off as required, but not turned on simultaneously. A 550 nm long pass filter, a band pass filter with center wavelengths of 632.8 ± 0.6 nm (FWHM: 3 ± 0.6 nm), 610 ± 2 nm, 620 ± 2 nm, 630 ± 2 nm, and 640 ± 2 nm (FWHM: 10 ± 2 nm) were used to select fluorescence at a particular wavelength. A polarization analyzer was used between the lens and BFP detector (Fig. 4c, 4d, 4g, 4h, 5d and Supplementary Figure 9d). The SEM

image of the dielectric multilayer and nanofibre are shown in the top right corner. The radius of the nanofibre was about 125 nm.

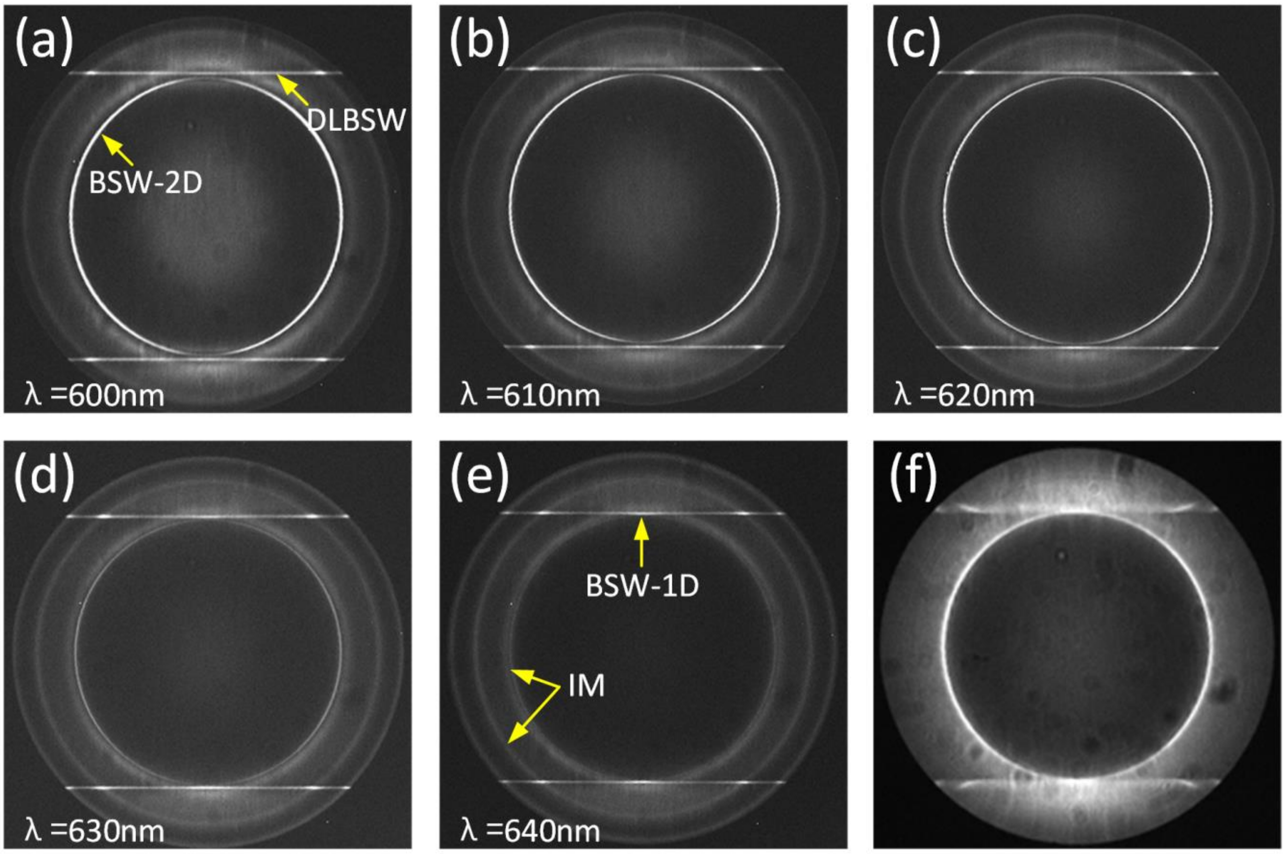

Supplementary Figure 6 | Evolution from DLBSW to BSW-1D with increasing wavelength. BFP fluorescence images of the nanofibre on the Multilayer-167 sample (the top SiO_2 layer had a thickness of about 167 nm). A series of band pass filters with center wavelengths of 600 nm (a), 610 nm (b), 620 nm (c), 630 nm (d), or 640 nm (e) were put in front of the camera to select the fluorescence wavelength; alternatively, a 550 nm long pass filter (f) was used. The FWHM of the band pass filter was 10 nm. The radius of the nanofibre, *R*, was about 100 nm. From (a) to (d), the bright ring (associated with the BSW-2D mode) and the lines appear simultaneously, which indicates the excitation of the DLBSW mode. In (e), only the bright lines appear which indicates the excitation of the BSW-1 D mode. The relatively weak rings associated with the internal mode (IM) of the

multilayer appear when using band-pass filters (a–e), but do not appear when using the 550 nm long pass filter (f).

Supplementary Note 1

Simulation of BFP image with FDTD. The fluorescence BFP images can also be numerically simulated with the finite-difference time-domain (FDTD) method, as shown in Fig. Supplementary Figure 7. Here, an electric dipole located in the middle of the nanofibre is used to mimic a single fluorescent molecule (similar to what is observed in Fig. 4i and 4j, where the focal point represents the location of the dipole). The dipole is oriented along the X-axis with an emission wavelength of 632.8 nm. The far-field distribution of the dipole emission is obtained from near- to far-field transformation according to surface equivalence theorem [39]. It is expected that the far-field angular distribution of the dipole emission is equivalent to the fluorescence distribution on the BFP. When t = 240 nm, we observe a split ring and two bright lines (Supplementary Figure 77a), and when t = 165 nm, we only observe a pair of bright lines (Supplementary Figure 77b), which are consistent with Figs. 4c and 4g, respectively. The dipole orientation and the polarizer direction (in Fig. 4c and 4g) are the same (both perpendicular to the long axis of the nanofibre, i.e., the X-axis).

When the dipole is oriented along the Z or Y direction, the bright lines cannot be excited efficiently owing to the TE or s-polarization property of the BSW (Supplementary Figure 8). These phenomena are also consistent with our experiment. In this experiment, the dye molecules have random orientations, meaning their electric dipoles also have random orientations. The BFP images without a polarizer before the detector contain significant fluorescence around the bright lines (Fig. 4b, 4f) owing to the randomly oriented dye molecules. This fluorescence around the bright lines

does not appear in the case of Fig. 4c and 4g where the polarizer was used to filter out the emission from dipoles oriented along the Y and Z directions (Supplementary Figure 8, Fig. 4d, 4h); this made the bright lines more distinct.

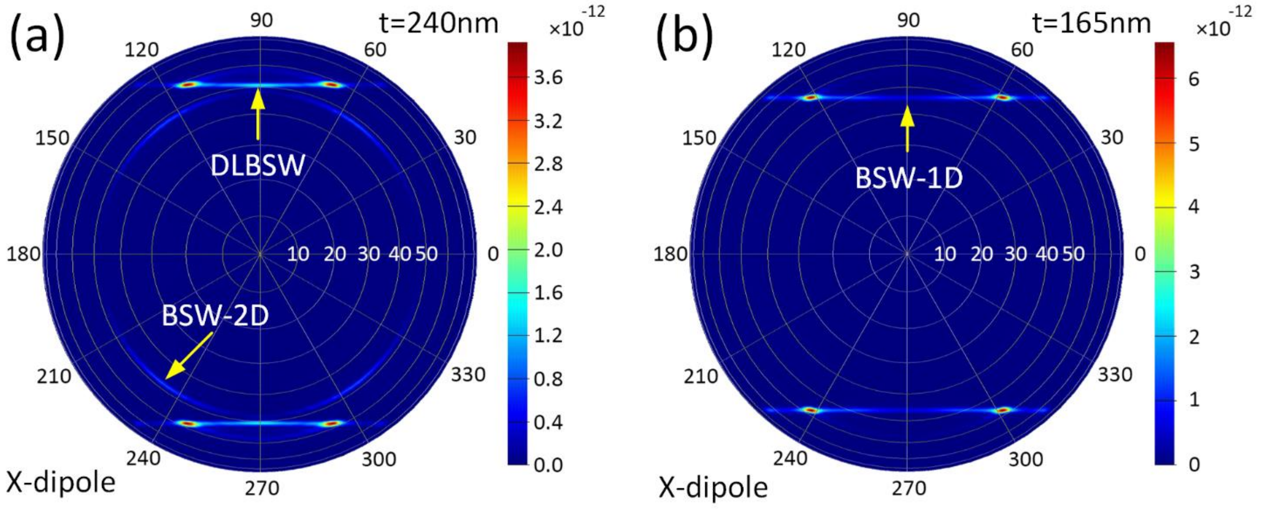

Supplementary Figure 7 | Simulated BFP images. An electronic dipole is put in the middle of the nanofibre with t = 240 nm (a) and t = 165 nm (b). The dipole is oriented along the X-axis (perpendicular to the long axis of the nanofibre). The emission wavelength is set as 632.8nm. The emission angle of the dipole into the glass substrate is θ (polar angle, 10, 20, 30, 40, 50, unit: degree in (a) and (b)) and ϕ (azimuthal angle, from 0 to 360, unit: degree in (a) and (b)). The angular dependent emission distribution is equivalent to the fluorescence distribution in the BFP image.

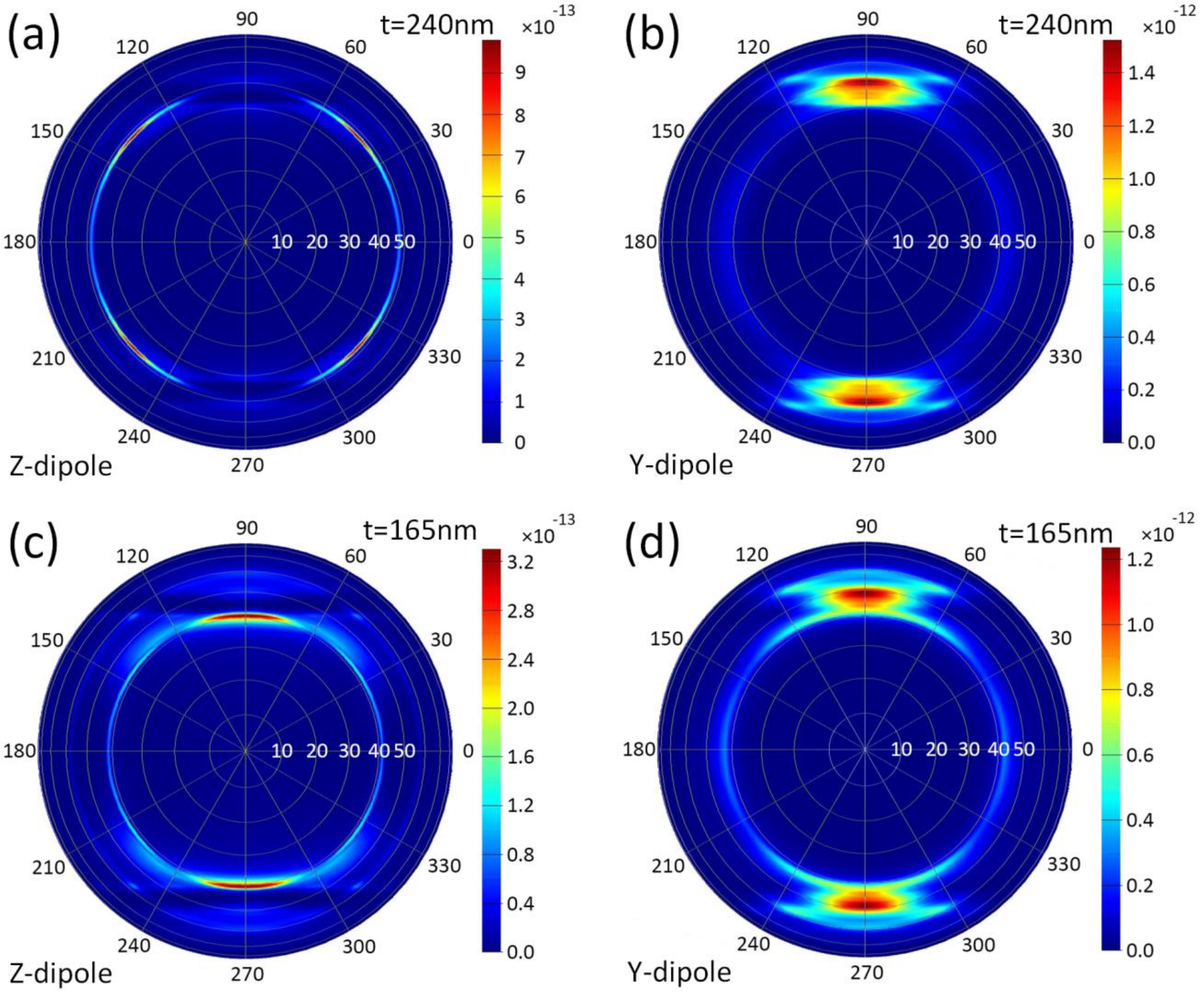

Supplementary Figure 8 | Simulated BFP images. An electronic dipole was located in the middle of a nanofibre with *t* =240 nm (a, b) and *t* = 165 nm (c, d). The dipole was oriented along the Z-axis (a, c) or along the Y-axis (b, d). The emission wavelength was set to 632.8 nm. The emission angle of the dipole into the glass substrate is described by θ (polar angle, 10, 20, 30, 40, 50, unit: degree in (a) and (b)) and ϕ (azimuthally angel, from 0 to 360, unit: degree in (a) and (b)). The angular dependent emission distribution is equivalent to the fluorescence distribution in the BFP image. No bright lines appeared in these images, which mean there was no excitation of either the DLBSW or BSW-1D mode.

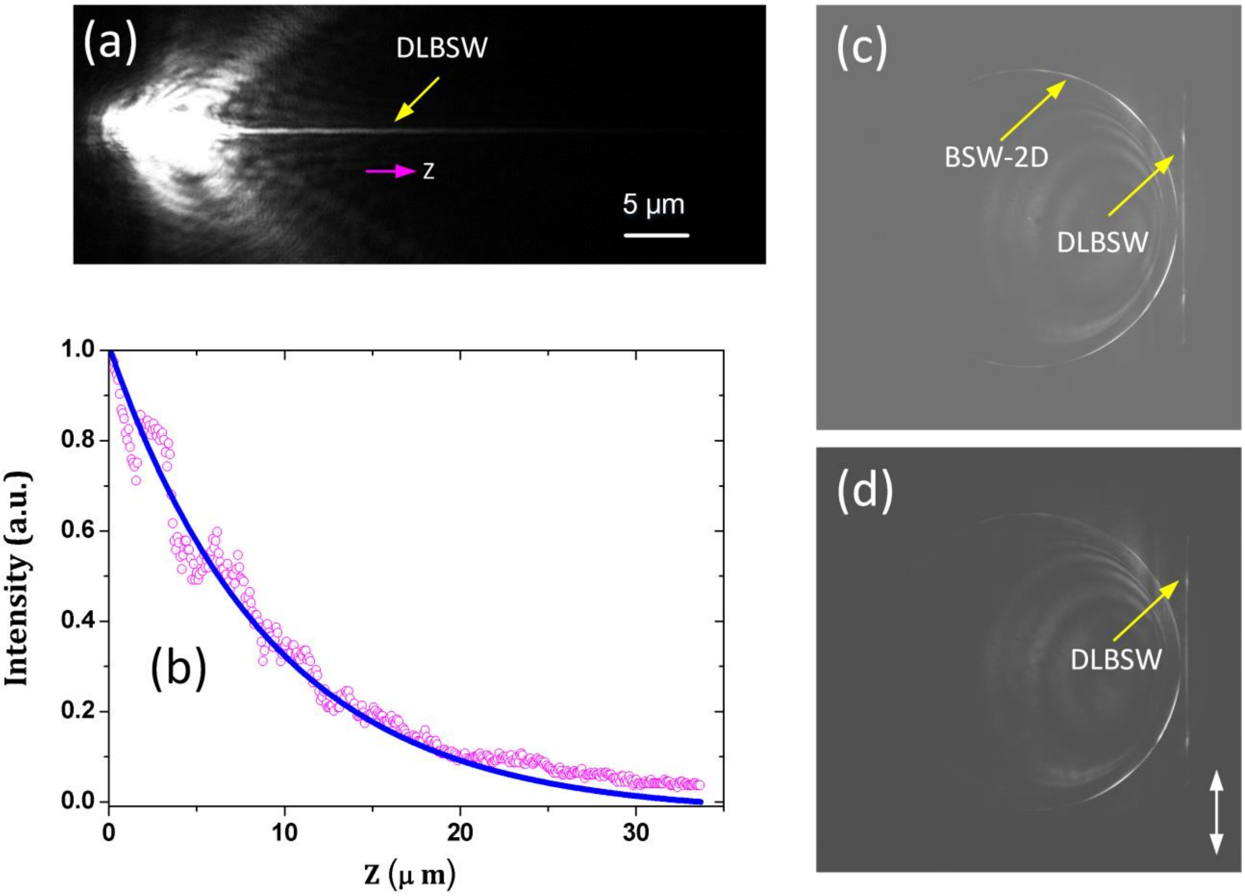

Supplementary Figure 9 | DLBSW and BSW-2D excited by laser beam from fibre tip. (a) FFP image of the laser beam propagating along the nanofibre. The laser beam has a wavelength of 632.8 nm. (b) Intensity distribution along the nanofibre (Z-direction, in (a)) is shown (red dots). The blue solid line is an exponential fit to the data and was used to extract the propagation length of the DLBSW as about 9 µm. Panels (c) and (d) show the corresponding BFP image with and without a polarizer before the detector. The double arrow-headed white line in (d) shows the polarizer orientation. Different from the Fig. 5, the BSW-2D (represented by the bright arcs) mode was also excited (on panel (c), (d)).
